# Quit Attempt Correlates among Smokers by Race/Ethnicity

**DOI:** 10.3390/ijerph8103871

**Published:** 2011-09-28

**Authors:** Jennifer W. Kahende, Ann M. Malarcher, Anna Teplinskaya, Kat J. Asman

**Affiliations:** 1 Centers for Disease Control and Prevention, National Center for Chronic Disease Prevention and Health Promotion, Office on Smoking and Health, 4770 Buford Highway, Atlanta, GA 30341, USA; E-Mails: amalarcher@cdc.gov (A.M.M.); ateplinskaya@cdc.gov (A.T.); 2 Research Triangle International, Statistics and Epidemiology Division, 4770 Buford Highway, Atlanta, GA 30341, USA; E-Mail: kasman@cdc.gov

**Keywords:** smoking, tobacco use, smoking cessation, smoking quit attempts

## Abstract

**Introduction:**

Cigarette smoking is the leading preventable cause of premature deaths in the U.S., accounting for approximately 443,000 deaths annually. Although smoking prevalence in recent decades has declined substantially among all racial/ethnic groups, disparities in smoking-related behaviors among racial/ethnic groups continue to exist. Two of the goals of Healthy People 2020 are to reduce smoking prevalence among adults to 12% or less and to increase smoking cessation attempts by adult smokers from 41% to 80%. Our study assesses whether correlates of quit attempts vary by race/ethnicity among adult (≥18 years) smokers in the U.S. Understanding racial/ethnic differences in how both internal and external factors affect quit attempts is important for targeting smoking-cessation interventions to decrease tobacco-use disparities.

**Methods:**

We used 2003 Tobacco Use Supplement to the Current Population Survey (CPS) data from 16,213 adults to examine whether the relationship between demographic characteristics, smoking behaviors, smoking policies and having made a quit attempt in the past year varied by race/ethnicity.

**Results:**

Hispanics and persons of multiple races were more likely to have made a quit attempt than whites. Overall, younger individuals and those with >high school education, who smoked fewer cigarettes per day and had smoked for fewer years were more likely to have made a quit attempt. Having a smoke-free home, receiving a doctor’s advice to quit, smoking menthol cigarettes and having a greater time to when you smoked your first cigarette of the day were also associated with having made a quit attempt. The relationship between these four variables and quit attempts varied by race/ethnicity; most notably receiving a doctor’s advice was not related to quit attempts among Asian American/Pacific Islanders and menthol use among whites was associated with a lower prevalence of quit attempts while black menthol users were more likely to have made a quit attempt than white non-menthol users.

**Conclusions:**

Most correlates of quit attempts were similar across all racial/ethnic groups. Therefore population-based comprehensive tobacco control programs that increase quit attempts and successful cessation among all racial/ethnic groups should be continued and expanded. Additional strategies may be needed to encourage quit attempts among less educated, older, and more addicted smokers.

## 1. Introduction

Cigarette smoking is the leading preventable cause of premature deaths in the U.S., accounting for approximately 443,000 deaths annually [1]. Although smoking prevalence in recent decades has declined substantially among all racial/ethnic groups [2,3], disparities in smoking-related behaviors among racial/ethnic groups continue to exist [2,4,5]. American Indians/Alaska Natives, blacks, and whites have higher smoking prevalence than Asian Americans/Pacific Islanders and Hispanics [2]. Regarding measures of smoking cessation, blacks and Hispanics are less likely to make successful quit attempts than whites [5,6]. Two of the goals of Healthy People 2020 are to reduce smoking prevalence among adults to 12% or less and to increase smoking cessation attempts by adult smokers from 41% to 80%. There are also goals designed to eliminate health disparities among racial/ethnic groups (*i.e.*, separate targets have been established for racial/ethnic groups) [7].

Studies have found that probability of making quit attempts and success of those attempts vary by demographic characteristics including gender, age and education [5,8]. Smoking behaviors (*i.e.*, amount of smoking, duration of smoking (years since initiation), level of nicotine dependence and type of cigarette smoked—menthol, non menthol) are also related to the probability of successful cessation, with those experiencing higher levels of dependence being less likely to quit [9]. Levels of dependence may vary by race/ethnicity; for example, one study found that African Americans were more likely to smoke within 30 minutes of waking than whites [10], while another found that whites were more likely to be heavier smokers than Hispanics and blacks [11]. Research also suggests that use of menthol cigarettes may impact smoking cessation [12,13]. Blacks and women are more likely to be menthol smokers than whites and men, and tobacco advertising efforts for such products are directed towards blacks and women [14–17]. Recently several studies have explored the relationship between menthol cigarettes and quit attempts among current smokers and found no relationship [18,19]. However, Fagan *et al*. also found that menthol smokers who reported smoking 6–10 cigarettes per day were more nicotine dependent than non-menthol smokers [18]. Other studies have found that menthol smokers are more likely to make quit attempts [13,20] but they are less successful at long term cessation [21,22]. The relationship between using menthol cigarettes and cessation also may vary by race/ethnicity with lower levels of quitting among blacks and Hispanics than among whites [22].

External factors are also associated with whether a smoker chooses to engage in a quit attempt, including receiving a doctor’s advice to quit, having smoke-free home rules, state/community smoke-free air policies, and increasing the price of cigarettes [23–25]. For example, smoke free air policies in work places often do not cover the service industry/sector and therefore those working in service industry (*i.e.*, restaurants and bars) are not receiving the benefits of these restrictions like those employed in other occupations where restrictions are in place [26]. Since minorities are more likely to be employed in the service industry it may be that workplace smoking restrictions vary more in particular racial/ethnic subgroups and may also have different variations with quit attempts in different groups. Also, blacks are more likely to smoke in the home and therefore smoke-free home rules may not be as important for these populations [26]. Smokers who receive advice to quit from their doctor are 30% more likely to quit than those who do not receive advice [25]. Significant racial and ethnic disparities in receiving advice to quit from health care professionals exist [27,28]. Black and Hispanic smokers are less likely to have been screened for tobacco use and advised to quit by health care professionals than white smokers [28,29]. Smoke-free home rules and workplace smoke-free policies seem to encourage smokers to quit or reduce their cigarette consumption [30–32]. These tobacco control policies vary by state and traditional tobacco-growing regions are less likely to have implemented these policies [33]. Our study examines whether sex, age, education, region, number of cigarettes smoked per day, how many years smoked, time to first cigarette, menthol, having smoking policy at work, having a home smoking rule, and receiving doctor’s advice to quit correlate with quit attempts among smokers from different racial/ethnic groups in the United States. Understanding racial/ethnic differences in how both internal and external factors affect quit attempts is important for targeting smoking-cessation interventions to decrease tobacco-use disparities. The Centers for Disease Control and Prevention through its National Tobacco Control Program seeks to eliminate disparities in tobacco use and can use these results for tailoring of educational materials and targeting of interventions to increase quit attempts. For example, if different relationships exist between particular age groups and cessation within different racial/ethnic groups then more educational efforts and targeting of cessation resources to those age and race/ethnic specific groups who are less likely to make quit attempts can be done to increase their interest in making a quit attempt.

## 2. Methods

We used the 2003 Tobacco Use Supplement to the Current Population Survey (TUS-CPS) data for our analysis (http://riskfactor.cancer.gov/studies/tus-cps/). CPS is a monthly survey conducted by the U.S. Bureau of the Census. The TUS-CPS is a national population based survey which is conducted every two years, includes approximately 50,000 households and collects information on respondents’ current and past smoking behaviors and demographic characteristics. The sample size for this study consisted of 16,213 adults (≥18 years of age) who had smoked within the past year. For our analysis, smokers were those who reported smoking at least 100 cigarettes during their lifetimes and smoked within the past year (includes those who remained smokers [currently smoked every day or some days] at the time of interview as well as former smokers at the time of interview [did not currently smoke] but who reported that they last smoked sometime during the past year). The following questions were used to assess whether a quit attempt was made in the past year. Current smokers at the time of interview who reported smoking on <12 days in the past 30 days were asked whether they had tried to quit smoking completely during the last 12 months. Current smokers at the time of interview who smoked >12 days in the past 30 days were asked if (1) they had ever stopped smoking for one day or longer because they were trying to quit and those who responded “yes” to that question were asked (2) whether during the past 12 months they had ever stopped smoking for one day or longer because they were trying to quit. Former smokers at time of interview reported on the length of time since they had completely quit smoking (if the length of time was during the past year they were classified as having a quit attempt during the past year).

We examined the prevalence of having a quit attempt in the past year among the following racial/ethnic groups; non-Hispanic whites, non-Hispanic blacks, Hispanics, American Asian/Pacific Islanders, American Indians/Alaska Natives, and non-Hispanic persons of more than one race (multi-race). We calculated the proportion of smokers who reported a quit attempt in the past year by race/ethnicity for other demographic variables, selected smoking characteristics and other related variables and calculated 95% confidence intervals for each proportion. These variables were gender, age, education, region of the country where he/she resides, amount smoked (*i.e.*, number of cigarettes smoked per day), duration of smoking (in years), an indicator of nicotine dependence (*i.e.*, how soon after waking do you smoke your first cigarette of the day?), brand of cigarette (menthol or non-menthol), the existence of workplace smoke-free policies, having home non-smoking rules, and receiving a doctor’s advice to quit within the past 12 month. Chi-squared tests were used to assess whether the proportion having a quit attempt varied across the characteristic’s levels. Data were then analyzed using multivariate logistic regression models to assess the independent effects of each of these variables on quit attempts as well as whether significant interactions occurred between these variables, race/ethnicity, and quit attempts.

For the multivariate logistic regression modeling, we included all of the above variables in the model as independent variables and their interaction terms with race/ethnicity. We then used backwards elimination regression modeling and eliminated all non-significant (statistically significant if p ≤ 0 .05 from the Wald statistic) interactions from t he initial model (the interaction with home smoke rule was borderline significant in the final model p = 0.09 but was retained because it was significant in some iterations of the model) and then one-by-one eliminated each of the non-significant main effects whose removal did not cause the beta coefficients of the other variables in the model to change. To account for weighting and the complex sampling design, we used SUDAAN 9.0.1 (Research Triangle Institute, Cary, North Carolina, USA) to calculate prevalence estimates, 95% confidence intervals.

## 3. Results

### 3.1. Study Population

Tables 1 and 2 show the proportion of smokers who had made a quit attempt during the past year among the six racial/ethnic groups by other demographic characteristics, specific smoking behaviors, and other external factors that may be related to quit attempts. Overall, 43.9% of smokers reported having made a quit attempt in the past year including 43.0% of whites, 44.9% of blacks, 47.6% of Hispanics, 48.0% of Asian Pacific Islanders, 48.3% of American Indian/American Natives, and 51.9% of persons of multiple races. Women (46.0%) were more likely to have made a quit attempt in the past year than men (42.1%) and the proportion of smokers who had made a quit attempt decreased with increasing age. Those with more than high school education were more likely to have made a quit attempt than those with less than high school education. Smokers living in the South were less likely to have made a quit attempt than smokers living in the other regions. Having had a quit attempt decreased as the amount smoked (number of cigarettes/day) and duration of smoking (number of years smoked) increased (Table 2). Also, the proportion of smokers who had a quit attempt tended to increase as time to first cigarette increased. No statistically significant differences in quit attempts were observed by type of cigarette smoked (menthol *vs*. non-menthol).

Smokers who were either not allowed to smoke indoors at work (46.6%) or who were employed where smoking was allowed at work (46.1%) had higher proportions of smokers who had made a quit attempt than smokers (41.9%) who were not employed in an indoor workplace (*i.e.*, employed but work primarily outside, home maker, retired, worked in a motor vehicle). Those who lived in households with a no smoking rule had a higher proportion of smokers who had made a quit attempt than those who lived in households without a no smoking rule. Those who received advice from a doctor to quit smoking had a higher proportion having made a quit attempts than those who did not. Whites (47.8%), blacks (50.0%), Hispanics (52.4%), and persons of multiple races (61.5%) who received advice to quit from a doctor were also more likely to quit than those who did not see a doctor (32.7%, 37.9%, 42.9%, and 38.2%, respectively) in the past 12 months.

For all variables except region, time to first cigarette, smoking policy at work, and doctor’s advice to quit, racial/ethnic subgroups had similar relationships between the characteristics examined and quit attempts as the overall relationships described above. For region, quit attempts among persons living in the South was not lower than persons living in other regions among American Indians and Alaska Natives and persons of multiple race (Table 1). Quit attempts increased consistently across categories of time to first cigarette among blacks (Table 2). Having no smoking allowed indoors at work was not significantly related to having made a quit attempt. Finally, receiving a doctor’s advice to quit was not associated with quit attempts among Asian Americans and Pacific Islanders or American Indians and Alaska Natives.

### 3.2. Multivariate Analysis

Our results in Table 3 show that odds of having made a quit attempt in the past year decreased with increasing age, and was lower among those who lived in the southern United States *vs*. those who lived in other areas. Current smokers with greater than a high school education (OR = 1.26, CI 1.15–1.38) were more likely to make a quit attempt than those with less than a high school education. The likelihood of making a quit attempt decreased as the number of cigarettes smoked per day increased. Current smokers who had smoked for 11–20 years (OR = 0.82, CI 0.71–0.93) and ≥20 years (OR = 0.79, CI 0.70–0.88) were less likely to make a quit attempt than current smokers who had smoked ≤5 years. Gender and smoking policy at work were not related to having made a quit attempt.

### 3.3. Interactions

Table 4 presents odds ratios for the significant interactions with race/ethnicity; race/ethnicity x home smoking rule, race/ethnicity x doctor’s advice to quit, race/ethnicity x time to first cigarette, race/ethnicity x menthol. Among all racial groups, having a home smoking rule was associated with a greater likelihood of having made a quit attempt. Among those without a smoke-free home rule, blacks and those of multiple race were more likely to have made a quit attempt than whites. Blacks who smoked menthol cigarettes (OR = 1.22, 95% CI 1.06–1.40) were more likely to make a quit attempt than whites who smoked non-menthol cigarettes. In contrast, whites who smoked menthol cigarettes (OR = 0.91, 95% CI 0.84–0.99) were less likely to make a quit attempt than whites who smoked non-menthol cigarettes. Persons of multiple races (OR = 1.42, 95% CI 1.13–1.78) who smoked non-menthol cigarettes were more likely to make a quit attempt than white non-menthol smokers. Whites (OR = 1.33, 95% CI 1.24–1.42), blacks (OR = 1.45, 95% CI 1.23–1.71), Hispanics (OR = 1.24, 95% CI 1.05–1.47), and persons of multiple races (OR = 2.25, 95% CI 1.59–3.19) who received doctor’s advice to quit were more likely to have made a quit attempt than whites who did not receive a doctor’s advice to quit. Physician advice to quit was not related to having made a quit attempt among Asian American/Pacific Islanders (OR = 1.04, 95% CI 0.72–1.52). While the odds ratio for receiving a doctor’s advice to quit was 1.42 (95% CI 0.94–2.15) for American Indians/Alaska Natives it was not statistically significant. In general those who smoked more than 30 minutes after waking were more likely to have made a quit attempt than whites who smoked their first cigarette less than 5 minutes after waking (this was statistically significant for all groups except American Indians/Alaska Natives—among this group the highest likelihood of having made a quit attempt was among those who smoked their first cigarette between 6 and 30 minutes after awakening.

## 4. Discussion

This study used a large population-based survey of U.S adults (≥18 years of age) to assess whether correlates of quit attempts among smokers vary by race/ethnicity. Our study found most correlates of quit attempts were similar across all racial/ethnic groups. We also observed that Hispanics and persons of multiple races were more likely to have made a quit attempt than whites. Although smoking prevalence continues to decline among all racial/ethnic groups, our findings, like those from earlier studies, show that disparities among racial/ethnic groups still exist in smoking-related behaviors including quit attempts with white smokers less likely to make a quit attempt than other racial/ethnic groups [2,4,5]. Studies have shown however, that among those who make a quit attempt, whites are more likely to be successful than other racial/ethnic groups [34].

Like previous studies [25,30,35], we found for all racial/ethnic subgroups that younger age, having greater than high school education, smoking fewer cigarettes, smoking for fewer years, not smoking within the first 5 minutes after waking, living in a home where smoking was not allowed indoors, and receiving a doctor’s advice to quit were positively associated with having made a quit attempt in the past year. Some of these correlates are aspects of a person’s smoking pattern and their level of nicotine dependence (*i.e.*, amount and duration of smoking and time to first cigarette). These correlates, as well as the relationship between younger age and increased quit attempts suggest that smokers who are younger and probably less nicotine dependent are the ones more likely to try to quit. Although young adult smokers are more likely to make a quit attempt than older adults, other studies have shown that they are less likely to use proven effective cessation treatments in their cessation attempts [5,36–38]. Findings from recent studies on the relationship between menthol cigarettes and smoking cessation have been mixed and although some have found no relationship or a negative relationship, it is important to note that most of those studies have looked at successful quitting, few stratified the relationships by race/ethnicity, and only four [13,18–20] have looked at the outcome of quit attempts. Alexander [19] and Cubbin [20] did not stratify by race/ethnicity and Alexander found no relationship with menthol and quitting while Cubbin found menthol smokers were not likely to quit. Levy *et al.* found that menthol smokers are more likely to make a quit attempt [13]. Levy *et al*. also found that menthol smokers were less successful at long term cessation, similar to Trinidad *et al.* [21]. We found that among whites, menthol users were less likely to make a quit attempt than non-menthol users. We also found that black menthol users were more likely to make a quit attempt than white menthol users. This may reflect the higher overall quit attempts among blacks *vs*. whites and the fact that most blacks smoke menthol cigarettes. More research is needed on the relationship between menthol use and cessation as studies to date have yielded conflicting results.

Studies have shown that cessation counseling and use of cessation medications increase smoking cessation [25,39,40]. Our study found that respondents who reported receiving doctor’s advice to quit (excluding Asian Americans/Pacific Islanders) were more likely to have made a quit attempt in the previous year than those who did not receive advice. Despite PHS clinical guidelines which recommend the delivery of effective cessation treatments for tobacco dependence at every clinical visit, disparities still exist in provider-delivered services [36,41–45]. Lopez-Quintero *et al*. found that about 16 million smokers had no recollection of receiving a physician’s advice to quit in the previous year [27]. They and Franks *et al*. [43] also found that blacks and Hispanics are less likely than whites to receive counseling in how to quit and the likelihood that Hispanics received counseling was not related to the smoker’s English language proficiency. Another important barrier to receiving counseling and effective medications is that not all smokers visit a health care provider each year; young smokers and blacks and Hispanics are less likely to see a physician.

One population-based strategy for providing counseling and medications is a toll-free quitline. Since 2006, all states have had a free tobacco cessation quitline; these quitlines provide a variety of effective smoking cessation services including counseling and can be accessed by calling 1-800-QUIT NOW. Quitlines have the potential to reach large numbers of smokers across all racial/ethnic populations and in recent years more smokers are accessing this service for smoking cessation assistance [46,47]. Quitlines are cost effective and they increase quit rates among callers by approximately 60% [25,48,49]. More research is needed to confirm whether Asian Americans/Pacific Islanders may not be benefiting from physician’s advice to quit as other racial/ethnic groups especially in light of recent research that indicate that although Asian smokers who spoke one of three Asian languages were just as likely as whites to call the California quitline, Asian smokers who spoke English were less likely to call the California quitline [50].

As evidence on the health effects of secondhand smoke continues to grow, the number of smoke-free laws and persons with voluntary rules aimed at protecting the family from secondhand smoke exposure in their home continues to grow. Although we did not find a relationship between workplace smoking policies and quit attempts in this study, laws that ban smoking indoors reduce opportunities to smoke and therefore reduce quantity of cigarettes consumed [51]. Clean indoor air laws may also positively increase the voluntary establishment of non-smoking rules in homes [52]. We found across all racial/ethnic groups that respondents living in a home in which smoking was not allowed indoors were more likely to have made a quit attempt than those who lived in homes where smoking was permitted (this relationship still persisted when persons who had already quit were excluded from the analysis to control for the possibility that the former smokers had changed their home smoking policy after they quit [data not shown]). Interestingly, a statistically significant interaction occurred between this variable and race/ethnicity and we observed that among those who did not have a smoke-free home Hispanics and Asian Americans/Pacific Islanders were no more likely to make a quit attempt than whites. Education campaigns encouraging the public in general to adopt smoke-free homes need to be implemented and advising smokers to adopt a smoke-free home should be included as part of cessation efforts by medical and public health practitioners [53].

One of the strengths of this survey is that it uses a nationally representative sample of the U.S. population which included large numbers of smokers within six racial/ethnic groups in the U.S. The survey, however, has some limitations: first, the TUS-CPS data were self-reported and data on past year quit attempts was collected retrospectively. Although, self-reported current cigarette smoking has good validity [54], reporting of quit attempts in the previous year depends on respondent’s recall of events and smokers may not accurately recall quit attempts of short duration [55]. Second, we were unable to assess causal relationships given that this was a cross-sectional study. However, many of our results were consistent with previous studies. Third, sample sizes for Asian American/Pacific Islanders, American Indian/Alaska Natives and persons of multiple races were small (n = 284–386) and therefore additional studies using larger samples are need to further confirm findings among these populations. Fourth, this study only examined the probability of making a quit attempt in the past year and not whether the quit attempt was successful. Future analyses are needed to examine whether patterns of successful cessation vary by race/ethnicity. Fifth, these racial/ethnic groups are heterogeneous and therefore we do not have information on correlates of quit attempts among smaller racial/ethnic groups such as those among subgroups of AA/PI (Koreans, Chinese, Samoans *etc*.). Sixth, questions on quit attempts in the past year differ slightly between current smokers who reported smoking on <12 days in the past 30 days and those who smoked ≥12 days in the past 30 days. In addition, non-white racial/ethnic groups are more likely to be light or infrequent smokers than whites [21,56]. Although we included a measure of amount of smoking in our model (number of cigarettes smoked per day) we did not control for number of days smoked in the past 30 days as it was highly correlated with number of cigarettes smoked per day. To examine whether question wording and frequency of smoking might have influenced our results, we re-ran the logistic regression models stratified by frequency of smoking (smoked <12 days *vs*. ≥12 days in the past 30 days) among current smokers (former smokers who quit in the past year were excluded). Because the numbers of smokers with each racial/ethnic group who reported smoking <12 days in the past 30 days was small the logistic regression model did not converge for this group. Among those who smoked ≥12 days in the past 30 days, the same variables that were statistically significant in our final logistic regression model for the entire populations were also significant among those who smoked ≥12 days in the past 30 days as were the interactions with race/ethnicity and doctor’s advice to quit and race/ethnicity and time to first cigarette and the race/ethnicity and menthol was borderline significant at p = 0.09. There was no longer an interaction with race/ethnicity and home smoking rules although home smoking rules was significant as a main effect in the model with those with home smoking rules more likely to make a quit attempt than those without home smoking rules. More research is needed on the effect of home smoking rules and the other correlates of quit attempts among infrequent smokers as this group is becoming a larger proportion of the population of smokers over time [57].

More research is needed to assess the effect of physician’s advice to quit on the probability of having a quit attempt among Asian American/Pacific Islanders as this variable was not related to having made a quit attempt in this group as in the other racial/ethnic groups. Because most correlates of quit attempts do not appear to vary substantially across racial/ethnic groups in the United States, comprehensive tobacco control programs that implement broad-based policy interventions (*i.e.*, smoke-free policies in public places, coverage for cessation treatment, increased price, mass media and pack labels, quitlines, *etc*.) should be effective in increasing quit attempts among all population racial/ethnic subgroups [58].

## Figures and Tables

**Table 1:** Proportion of smokersΦ who reported a quit attempt in the past year by race/ethnicity and other demographic characteristics.

	White§ % (CI)	Black§ % (CI)	Hispanic % (CI)	AA/PI§Ψ % (CI)	AI/AN§ρ % (CI)	Multipleχχ % (CI)	Total % (CI)
Total sample size = 16,213	12,794	1,310	1,129	310	284	386	16,213
Total	43.0 (42.2–43.8)	44.9 (42.6–47.2)	47.6 (45.4–49.7)	48.0 (43.8–52.3)	48.3 (42.0–54.7)	51.9 (47.5–56.2)	43.9 (43.3–44.6)
**Gender**							
Male	41.0 (40.0–42.0)	43.1 (40.2–46.0)	46.7 (43.7–49.8)	45.9 (40.6–51.2)	46.6 (37.3–56.2)	48.1 (41.7–54.5)	42.1 (41.3–43.0)
Female	45.2 (44.1–46.4)	46.8 (43.7–50.0)	49.2 (45.6–52.7)	53.4 (46.2–60.5)	50.0 (42.1–58.0)	56.0 (50.2–61.7)	46.0 (45.0–47.0)
**Age (years)**							
18–24	53.4 (51.4–55.5)	51.0 (43.7–58.3)	51.4 (46.1–56.7)	54.3 (39.3–68.5)	n/a	58.2 (47.8–67.9)	53.1 (51.5–54.8)
25–34	48.0 (46.2–49.8)	51.4 (45.8–57.0)	50.2 (45.2–55.2)	49.2 (41.9–56.6)	50.2 (37.9–62.4)	53.2 (43.5–62.6)	48.8 (47.3–50.3)
35–44	42.2 (40.8–43.6)	44.3 (40.2–48.3)	49.7 (45.7–53.7)	52.5 (42.4–62.3)	55.6 (45.4–65.4)	45.9 (36.2–56.0)	43.6 (42.3–44.8)
45–64	37.1 (35.9–38.2)	40.3 (37.1–43.6)	42.4 (38.4–46.6)	40.7 (31.5–50.7)	40.2 (31.3–49.9)	49.7 (42.5–56.9)	38.1 (37.0–39.2)
65+	37.3 (35.1–39.5)	41.4 (34.2–48.8)	28.8 (20.2–39.2)	n/a	n/a	n/a	37.5 (35.5–39.5)
**Education**							
<High School	36.5 (34.6–38.5)	42.3 (38.3–46.4)	46.3 (42.1–50.5)	34.7 (22.5–49.3)	42.2 (30.9–54.3)	46.4 (35.4–57.8)	39.5 (37.9–41.2)
HS/GED	40.3 (39.2–41.5)	41.7 (38.1–45.3)	47.3 (43.0–51.7)	40.5 (32.0–49.6)	49.1 (40.1–58.2)	46.3 (37.7–55.1)	41.1 (40.1–42.2)
>High School	47.6 (46.5–48.7)	50.6 (47.0–54.1)	49.4 (44.9–54.0)	53.3 (48.3–58.2)	51.7 (41.3–61.9)	58.3 (52.1–64.1)	48.4 (47.4–49.4)
**Region**							
Northeast	45.4 (43.5–47.2)	45.6 (40.8–50.4)	49.8 (43.2–56.5)	47.2 (35.9–58.7)	n/a	48.2 (33.7–63.1)	45.9 (44.1–47.8)
Midwest	44.4 (43.0–45.8)	46.7 (42.5–50.8)	49.7 (43.6–55.8)	51.7 (40.0–63.2)	44.8 (34.6–55.5)	50.7 (41.4–60.0)	45.0 (43.8–46.2)
South	39.3 (37.8–40.8)	42.5 (39.5–45.6)	44.8 (41.2–48.6)	47.2 (37.0–57.6)	44.9 (36.8–53.3)	51.6 (45.1–58.0)	40.6 (39.4–41.8)
West	46.1 (44.3–47.9)	52.5 (44.2–60.7)	48.8 (45.1–52.4)	47.7 (40.7–54.8)	47.7 (36.4–59.1)	54.1 (46.7–61.4)	47.2 (45.9–48.6)

Φ Smokers were those who smoked at least 100 cigarettes during their lifetime and, smoked within the past year.

§ Non-Hispanic;

Ψ Asian American/Pacific Islander;

ρ American Indian/Alaska Native;

χχ Multiple Races.

Note: Chi-squared tests for total group were statistically significant p ≤ 0.05 for all variables. n/a—small sample size.

**Table 2:** Proportion of SmokersΦ who reported a quit attempt in the past year by race/ethnicity and selected smoking characteristics.

	White§ % (CI)	Black§ % (CI)	Hispanic % (CI)	AA/PI§Ψ % (CI)	AI/AN§Ф % (CI)	Multiple§χχ % (CI)	Total % (CI)
**Number of cigarettes per day**							
<5	52.9 (50.8–54.9)	55.1 (50.3–59.9)	46.2 (42.7–49.6)	49.8 (41.3–58.3)	n/a	54.2 (43.2–64.8)	51.8 (50.4–53.2)
5–14	51.0 (49.7–52.3)	43.0 (40.1–45.9)	48.4 (44.5–52.4)	52.3 (45.0–59.5)	55.5 (44.4–66.1)	54.7 (46.5–62.7)	49.6 (48.4–50.9)
15–34	37.9 (36.7–39.1)	39.5 (35.5–43.7)	39.4 (35.0–44.0)	36.0 (27.4–45.7)	35.6 (27.0–45.2)	48.0 (40.0–56.1)	38.2 (37.2–39.3)
25+	29.7 (28.0–31.5)	31.8 (22.7–42.5)	41.1 (28.8–54.6)	n/a	n/a	37.7 (27.4–49.2)	30.3 (28.7–31.9)
**How long smoked**							
≤5 years	55.6 (52.9–58.1)	55.5 (47.6–63.2)	52.5 (45.4–59.6)	57.4 (42.3–71.2)	n/a	59.6 (45.0–72.7)	55.5 (53.2–57.6)
6–10 years	52.0 (49.7–54.3)	51.3 (44.9–57.6)	53.5 (46.7–60.2)	57.4 (45.2–68.7)	n/a	60.3 (48.7–70.8)	52.4 (50.3–54.4)
11–20 years	46.2 (44.5–47.9)	47.7 (42.0–53.6)	48.2 (43.7–52.8)	46.0 (39.0–53.1)	51.2 (35.5–66.7)	50.3 (39.7–60.7)	46.7 (45.2–48.2)
20+ years	37.9 (37.0–38.9)	41.3 (38.4–44.2)	44.0 (41.1–47.0)	44.5 (35.8–53.5)	45.7 (39.3–52.2)	48.1 (41.1–55.2)	39.0 (38.1–39.9)
**Time to 1^sst^ Cigarette**							
≤5 min	33.2 (31.9–34.5)	47.1 (42.0–52.3)	40.5 (34.2–47.2)	43.8 (30.9–57.6)	32.1 (20.9–45.8)	41.2 (32.4–50.7)	35.4 (34.2–36.8)
6–30 min	37.3 (36.1–38.6)	40.0 (35.6–44.6)	41.3 (35.9–46.9)	36.7 (28.0–46.4)	55.4 (44.0–66.2)	43.5 (35.9–51.4)	38.0 (36.9–39.1)
31–60 min	47.5 (45.9–49.2)	43.4 (39.0–47.9)	51.0 (45.1–56.9)	58.0 (48.9–66.5)	51.7 (37.3–65.8)	63.1 (53.0–72.5)	47.9 (46.5–49.3)
61+ min	56.7 (55.2–58.1)	54.7 (50.1–59.2)	52.1 (48.6–55.5)	54.5 (44.4–64.2)	51.1 (40.1–61.9)	62.3 (52.6–71.2)	55.7 (54.5–56.9)
**Menthol**							
No	43.4 (42.6–44.2)	41.2 (36.5–46.0)	47.2 (44.7–49.6)	49.2 (44.4–54.1)	47.7 (40.4–55.2)	51.9 (47.2–56.5)	44.0 (43.2–44.7)
Yes	41.8 (39.9–43.7)	47.2 (44.9–49.6)	51.7 (47.3–56.1)	53.5 (44.8–61.9)	52.6 (38.9–65.9)	52.0 (41.9–62.0)	44.7 (43.4–46.1)
**Smoking policy at work**							
Smoking allowed indoors at work	45.5 (43.7–47.3)	48.9 (43.5–54.3)	48.9 (43.3–54.4)	36.6 (35.0–58.7)	n/a	42.3 (32.2–53.2)	46.1 (44.6–47.6)
No smoking allowed indoors at work	45.9 (44.5–47.3)	44.3 (39.8–48.9)	50.9 (46.1–55.8)	55.8 (47.9–63.4)	53.2 (42.9–63.3)	53.4 (44.9–61.7)	46.6 (45.4–47.9)
Does not work indoors	40.7 (39.8–41.8)	44.3 (41.5–47.2)	45.8 (43.1–48.4)	42.4 (36.3–48.7)	35.8 (38.2–53.7)	52.3 (46.6–58.0)	41.9 (41.1–42.7)
**Home smoking rules**							
Smoking allowed at home	35.6 (34.6–36.6)	42.2 (39.7–44.8)	39.7 (36.5–43.0)	34.3 (27.9–41.2)	41.2 (33.9–48.9)	33.1 (37.3–49.0)	36.8 (35.9–37.7)
Smoking not allowed at home	55.5 (54.1–56.8)	52.0 (47.4–56.5)	53.6 (50.4–56.7)	57.3 (51.4–63.0)	61.0 (50.5–70.5)	66.4 (58.7–73.3)	55.3 (54.1–56.4)
**Received Doctor advice to quit**							
Yes	47.8 (46.6–48.9)	50.0 (46.8–53.2)	52.4 (48.7–56.0)	53.0 (44.8–61.1)	53.2 (43.2–62.9)	61.5 (54.5–68.0)	48.6 (47.6–49.7)
No	44.5 (43.1–45.9)	45.1 (40.6–49.6)	50.3 (46.1–54.4)	52.6 (45.3–59.8)	58.6 (48.1–68.3)	49.9 (42.9–56.8)	45.4 (44.1–46.7)
Didn’t see Doctor past 12 month	32.7 (31.3–34.2)	37.9 (34.5–41.5)	42.7 (38.7–46.8)	40.5 (33.4–48.0)	34.9 (25.5–45.6)	38.2 (30.4–46.6)	35.1 (33.8–36.5)

Φ Smokers were those who smoked at least 100 cigarettes during their lifetime and, smoked within the past year.

§ Non-Hispanic;

Ψ Asian American/Pacific Islander;

Ф American Indian/Alaska Native;

χχ Multiple Races.

Note: Chi-squared tests for total group were statistically significant p ≤ 0.05 for all variables. n/a—small sample size.

**Table 3:** Adjusted odds ratios from final multivariate logistic regression model Ж for correlates of having a quit attempt in the past year among smokers Φ.

	Odds Ratios	Confidence Interval (95%)
**Gender**		
Male	1.00	Ref
Female	1.05	1.00–1.12
**Age (years)**		
18–24	1.00	Ref
25–34	0.94	0.86–1.04
35–44	0.93	0.82–1.04
45–64	0.78*	0.68–0.88
≥65	0.71*	0.61–0.84
**Education**		
Less than High School	1.00	Ref
High School Diploma/GED	1.06	0.98–1.15
Greater than High School	1.26*	1.15–1.38
**Region**		
Northeast	1.00	Ref
Midwest	1.04	0.94–1.14
South	0.85*	0.77–0.94
West	0.95	0.86–1.05
**Number of cigarettes per day**		
<5	1.00	Ref
5–14	0.94	0.86–1.03
15–24	0.72*	0.65–0.79
≥25	0.59*	0.52–0.67
**How long smoked**		
≤5 years	1.00	Ref
6–10 years	0.98	0.85–1.14
11–20 years	0.82*	0.71–0.93
20+ years	0.79*	0.70–0.88
**Smoking policy at work**		
Smoking allowed indoors at work	1.00	Ref
Smoking not allowed indoors at work	0.95	0.87–1.03
Does not work indoors	0.98	0.91–1.06

Ж Includes all variables in the table plus main effects for race/ethnicity and home smoking rule, doctor’s advice to quit, time to first cigarette, menthol and interactions of race/ethnicity by home smoking rule, doctor’s advice to quit, time to first cigarette, and menthol (see Table 4 for interactions).

Φ Smokers were those who smoked at least 100 cigarettes during their lifetime and smoked within the past year.

* p ≤ 0.05.

**Table 4:** Adjusted odds ratios for statistically significant interactions with race/ethnicity from multivariate logistic regression model ^Ж^ for having a quit attempt in the past year among smokers.

	White§	Black§	Hispanic	AA/PI§Ψ	AI/AN§Ф	Multiple§χχ
**Home smoking rule**						
Smoking allowed	Ref	1.28* (1.11–1.47)	1.07 (0.92–1.25)	0.88 (0.64–1.21)	1.13 (0.81–1.59)	1.33* (1.03–1.71)
Smoking not allowed	1.79* (1.65–1.94)	1.63* (1.32–2.01)	1.63* (1.40–1.90)	1.85* (1.40–2.45)	2.13* (1.32–3.44)	2.52* (1.73–3.68)
**Received Doctor Advice to quit**						
No	Ref	1.07 (0.85–1.34)	0.94 (0.79–1.12)	1.10 (0.76–1.57)	1.93* (1.27–2.94)	1.00 (0.72–1.38)
Yes	1.33* (1.24–1.42)	1.45* (1.23–1.71)	1.24* (1.05–1.47)	1.04 (0.72–1.52)	1.42 (0.94–2.15)	2.25* (1.59–3.19)
Didn’t see doctor past 12 months	0.70* (0.65–0.76)	1.00 (0.84–1.18)	0.76* (0.62–0.93)	0.78 (0.56–1.10)	0.59 (0.34–1.01)	0.92 (0.62–1.36)
**Time to first cigarette**						
≤5 minutes	Ref	1.70* (1.36–2.13)	1.25 (0.96–1.64)	1.30 (0.74–2.27)	0.78 (0.39–1.53)	1.45 (0.97–2.15)
6–30 minutes	1.08 (1.00–1.17)	1.19 (0.95–1.49)	1.20 (0.94–1.54)	0.83 (0.54–1.27)	2.31* (1.46–3.64)	1.37 (0.98–1.91)
31–60 minutes	1.37* (1.25–1.51)	1.24 (0.99–1.55)	1.49* (1.16–1.93)	1.75* (1.18–2.60)	1.51 (0.85–2.67)	2.41* (1.52–3.82)
>60 minutes	1.50* (1.34–1.66)	1.69* (1.33–2.15)	1.26* (1.05–1.51)	1.28 (0.86–1.92)	1.38 (0.92–2.07)	1.63* (1.02–2.60)
**Menthol**						
No	Ref	1.01 (0.79–1.29)	0.94 (0.83–1.06)	0.94 (0.74–1.20)	1.09 (0.79–1.50)	1.42* (1.13–1.78)
Yes	0.91* (0.84–0.99)	1.22* (1.06–1.40)	1.08 (0.87–1.33)	1.18 (0.77–1.79)	1.26 (0.66–2.40)	1.10 (0.69–1.75)

Final model also includes all variables in Table 3 and main effects for race/ethnicity and home smoking rule, doctor’s advice to quit, time to first cigarette, and menthol.

Φ Smokers were those who smoked at least 100 cigarettes during their lifetime and smoked within the past year.

§ Non-Hispanic;

Ψ Asian American/Pacific Islander;

Ф American Indian/Alaska Native;

χχ Multiple Races.

* p ≤ 0.05.
